# ALKBH5-mediated N^6^-methyladenosine modification of TRERNA1 promotes DLBCL proliferation via p21 downregulation

**DOI:** 10.1038/s41420-022-00819-7

**Published:** 2022-01-14

**Authors:** Wei Song, Fei Fei, Fengchang Qiao, Zuyi Weng, Yuanxun Yang, Bei Cao, Jing Yue, Jiaxuan Xu, Meihong Zheng, Juan Li

**Affiliations:** 1 Phase I Clinical Trials Unit, Nanjing Drum Tower Hospital, The Affiliated Hospital of Nanjing University Medical School, Nanjing, 210008 China; 2 Anhui Province Key Laboratory of Translational Cancer Research (Bengbu Medical College), Bengbu, 233030 China; 3 Women’s Hospital of Nanjing Medical University, Nanjing Maternity and Child Health care Hospital, Nanjing, 210001 China; 4 Department of Gynaecology and Obstetrics, Nanjing Drum Tower Hospital, The Affiliated Hospital of Nanjing University Medical School, Nanjing, 210008 China

**Keywords:** Tumour biomarkers, Genetics research, Long non-coding RNAs, Gene silencing, Prognostic markers

## Abstract

Long noncoding RNAs (lncRNAs) have crucial functions in the tumorigenesis and metastasis of cancers. N^6^-methyladenosine (m^6^A) modification of RNA is an important epigenetic regulatory mechanism in various malignancies. Nevertheless, the mechanism of m^6^A-modified lncRNA in diffuse large B cell lymphoma (DLBCL) has remained poorly defined. In the present study, we showed that lncRNA TRERNA1 was associated with the poor prognosis of DLBCL patients. TRERNA1 with internal m^6^A modification was highly correlated with the demethylase ALKBH5 expression. We further demonstrated that TRERNA1 was a potential downstream target of ALKBH5-mediated m^6^A modification by m^6^A-RNA sequencing and m^6^A-RIP assays. Decreased m^6^A methylation of TRERNA1 regulated by ALKBH5 was shown to regulate cell proliferation in vitro and in vivo. The results of mechanism analyses revealed that TRERNA1 recruited EZH2 to epigenetically silence the expression of the cyclin-dependent kinases inhibitor p21 by H3K27me3 modification of its promoter region. In addition, ALKBH5 further inhibited p21 expression. Taken together, our results elucidate the functional roles and epigenetic alterations of TRERNA1 through m^6^A modification in DLBCL. TRERNA1, the expression of which is upregulated by ALKBH5, acts as a scaffold that decreases p21 expression. The results of the present study provide novel targets for the diagnosis and treatment of DLBCL.

## Introduction

Diffuse large B cell lymphoma (DLBCL) is one of the most common hematologic malignancies and exhibits a striking degree of genetic heterogeneity worldwide [1]. At present, rituximab, cyclophosphamide, doxorubicin, vincristine, and prednisone (R-CHOP) are widely used in the majority of DLBCL patients, yet 30–40% of patients exhibit poor outcomes from the standard treatment [2, 3]. Advances in molecular genetics have vastly improved the understanding of the biological diversity of DLBCL. In the past decade, numerous epigenetic modifiers have been used for the clinical treatment of patients with hematologic malignancies, including inhibitors of DNA methyltransferases (DNMTs), enhancers of zeste homolog 2 (EZH2), histone deacetylases (HDACs), and isocitrate dehydrogenases (IDHs) [4]. However, because few studies have investigated the epigenetic mechanism of DLBCL, it is important to identify epigenetic molecular targets and predictive biomarkers for the treatment of DLBCL.

Long noncoding RNAs (lncRNAs) are a novel class of gene regulators that are defined as transcripts longer than 200 nucleotides without protein-coding capacity [5]. LncRNAs function as scaffolds, signals, guides, and decoys via DNA, RNA, protein, or long-range chromatin interactions [6, 7]. Accumulating evidence has shown that lncRNAs have a broad range of tumorigenesis and metastasis functions as key regulators in a variety of cancers [8]. LncRNAs have prognostic value in DLBCL patients, and the expression patterns of lncRNAs can characterize distinct stages of B-cell development and activation [9, 10]. Translational regulatory lncRNA 1 (TRERNA1) was first reported as an enhancer-like RNA in mediating the expression of its neighboring genes [11]. TRERNA1 is positively correlated with lymph node metastasis, and its expression stimulates the invasion and metastasis of breast and gastric cancer [12, 13]. The results of our previous study also revealed that TRERNA1 promotes hepatocellular carcinoma (HCC) metastasis via recruitment of the EHMT2/SNAI1 complex to suppress CDH1 [14]. However, little is known regarding the TRERNA1-mediated regulation of DLBCL malignant progression.

N^6^-methyladenosine (m^6^A), the most prevalent RNA methylation modification, regulates gene expression by altering RNA splicing, editing, stability, degradation, and lncRNA/circular RNA activity [7, 15–17]. Increasing evidence has shown that m^6^A modification significantly affects the pathogenesis of multiple cancers, with recent studies, have shown that m^6^A mRNA methylation plays an important role in regulating T cell homeostasis and the carcinogenesis of acute myeloid leukemia [18, 19]. Thus, it is important to elucidate the regulatory mechanisms associated with m^6^A RNA methylation in DLBCL.

In the present study, we investigated the potential function of TRERNA1 in the progression of DLBCL. TRERNA1 promotes the tumorigenesis of DLBCL and is a target of ALKBH5 (α-ketoglutarate-dependent dioxygenase alkB homolog 5), a RNA demethylase that modifies m^6^A methylation. Further characterization revealed that lower m^6^A modification of TRERNA1 suppresses p21 expression via the recruitment of EZH2. Our results reveal the important role of TRERNA1 in DLBCL and show that m^6^A modification of this lncRNA may serve as a promising marker of this disease.

## Results

### Upregulated lncRNA TRERNA1 expression is negatively correlated with m^6^A modification

**Fig. 1:**
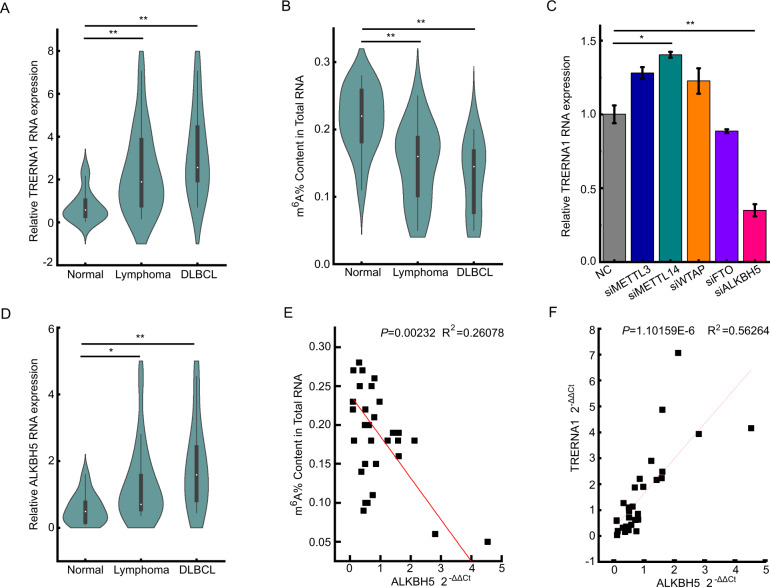
TRERNA1 is highly expressed in DLBCL and is associated with m^6^A modification. **A** TRERNA1 RNA expression in DLBCL tissues and normal lymph node hyperplasia tissues. **B** The m^6^A contents of total RNA from 15 lymphoma tissues (including 8 cases of diffuse large B cell lymphoma) and 15 normal lymph node hyperplasia tissues. **C** TRERNA1 expression levels were measured by RT-qPCR after transfection with different m^6^A modification-associated genes. **D** The ALKBH5 expression in DLBCL tissues and normal lymph node hyperplasia tissues. **E** The correlation between ALKBH5 levels and m^6^A in DLBCL. **F** Scatter plot between ALKBH5 and TRERNA1 in DLBCL. The data are presented as the mean ± SD values; *n* = 3. *P < 0.05, **P < 0.01.

LncRNAs are key epigenetic molecules that affect development and disease progression and have a profound impact on the regulation of a variety of tumors, including lymphoma. The results of our previous study demonstrated that lncRNA TRERNA1 is closely associated with lymph node metastasis in gastric cancer, breast cancer, and HCC. To investigate the potential function of TRERNA1 in DLBCL progression, we assessed the levels of TRERNA1 expression in 15 normal lymph node hyperplasia tissues and 15 lymphoma tissues (including 8 cases of diffuse large B cell lymphoma). The RT-qPCR results showed that TRERNA1 expression levels were higher in DLBCL tissues than in the control tissues (Fig. 1A). m^6^A modification has an important role in regulating gene expression and may be associated with human cancers [20]. To investigate the potential role of m^6^A modification in lymphoma, we first examined the levels of m^6^A in total RNA samples using a colorimetric m^6^A quantification approach. We observed that m^6^A levels were lower in DLBCL tissues than in normal lymph node hyperplasia tissues (Fig. 1B). Next, we evaluated whether m^6^A modifying enzyme can regulate the expression of TRERNA1 (Fig. 1C and Fig. S1A). We observed that the knockdown of ALKBH5, a key m^6^A eraser, decreased TRERNA1 expression. In addition, we assessed the clinical expression of ALKBH5 in DLBCL by RT-qPCR, the results of which showed that ALKBH5 was more highly expressed in DLBCL tissues than in normal hyperplasia tissues (Fig. 1D). Furthermore, a negative correlation was observed between m^6^A and ALKBH5 RNA levels (Fig. 1E), and we also observed a positive and significant relationship between ALKBH5 and TRERNA1 expression levels (Fig. 1F). These data indicated that the negative regulation of TRERNA1 by m^6^A modification might be a potential prognostic indicator for DLBCL.

### ALKBH5-mediated TRERNA1 regulation promotes cell proliferation and cell cycle progression in DLBCL

**Fig. 2:**
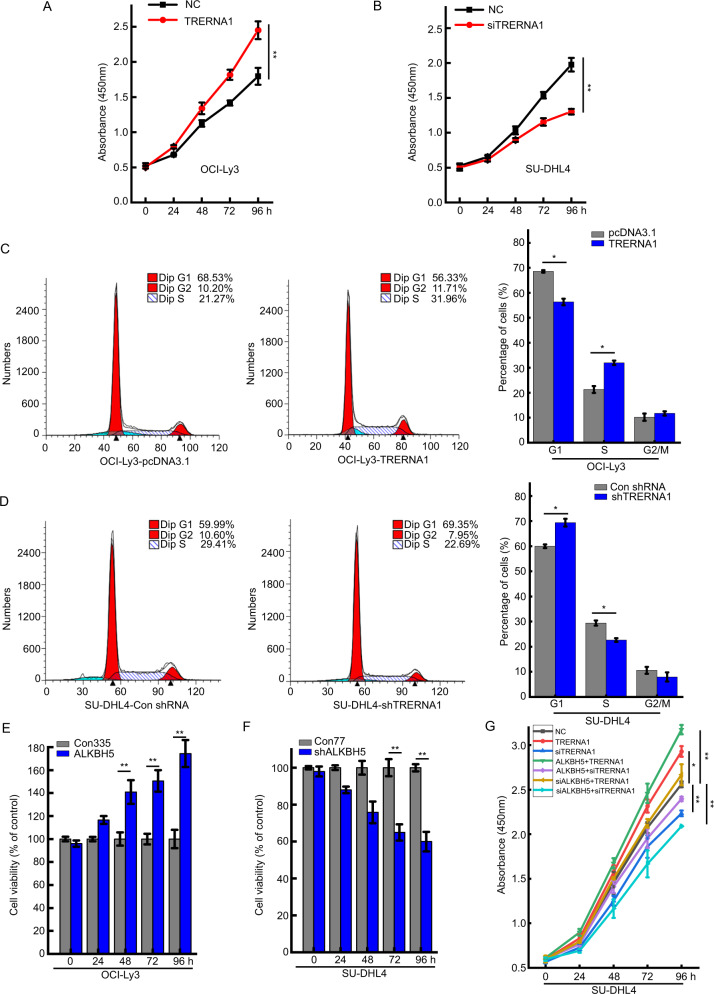
ALKBH5-mediated regulation of TRERNA1 promotes cell proliferation and cell cycle progression in DLBCL. **A** TRERNA1 overexpression promoted cell proliferation, as determined by the CCK-8 assay. **B** TRERNA1 silencing suppressed cell proliferation, as assessed by CCK-8 assay. **C** TRERNA1 accelerated cell cycle progression at the G1/S phase in OCI-Ly3 cells as determined by flow cytometry. **D** TRERNA1 silencing induced cell cycle arrest at G1/S phase in SU-DHL4 cells, as assessed by flow cytometry. **E** ALKBH5 upregulation enhanced the viability of OCI-Ly3 cells (Con335 is the negative control group relative to TRERNA1 overexpression). **F** ALKBH5 silencing decreased the viability of SU-DHL4 cells (Con77 is the negative control group relative to TRERNA1 knockdown). **G** Cell proliferation was assessed after the indicated transfections. The data are presented as the mean ± SD values; *n* = 3. *P < 0.05, **P < 0.01.

To examine whether TRERNA1 is involved in DLBCL development, we overexpressed TRERNA1 in OCI-Ly3 cells, the results of which showed that cell proliferation was higher in TRERNA1-overexpressing than the empty vector group according to the results in CCK-8 assays (Fig. 2A and Fig. S2A). TRERNA1 overexpression promoted cell cycle progression as determined by flow cytometry analysis (Fig. 2C). In addition, TRERNA1 knockdown in SU-DHL4 cells resulted in reduced cell proliferation compared to that observed in the control as assessed by CCK-8 assays (Fig. 2B). Furthermore, TRERNA1 knockdown notably promoted cell cycle arrest at the G1/S phase (Fig. 2D). Given the observed relationship between ALKBH5 and TRERNA1 in DLBCL tissues, we subsequently assessed whether the function of TRERNA1 is influenced by ALKBH5. We observed that ALKBH5 upregulation promoted cell viability, while cell viability was reduced when ALKBH5 expression was knocked down (Fig. 2E, F). ALKBH5 upregulation significantly reversed the inhibition of cell proliferation induced by TRERNA1 silencing. Moreover, ALKBH5 knockdown markedly abolished the increased cell proliferation induced by TRERNA1 overexpression (Fig. 2G). These results demonstrated that TRERNA1 upregulation by ALKBH5 promoted cell proliferation and cell cycle progression.

### ALKBH5 upregulates TRERNA1 expression via m^6^A modification

**Fig. 3:**
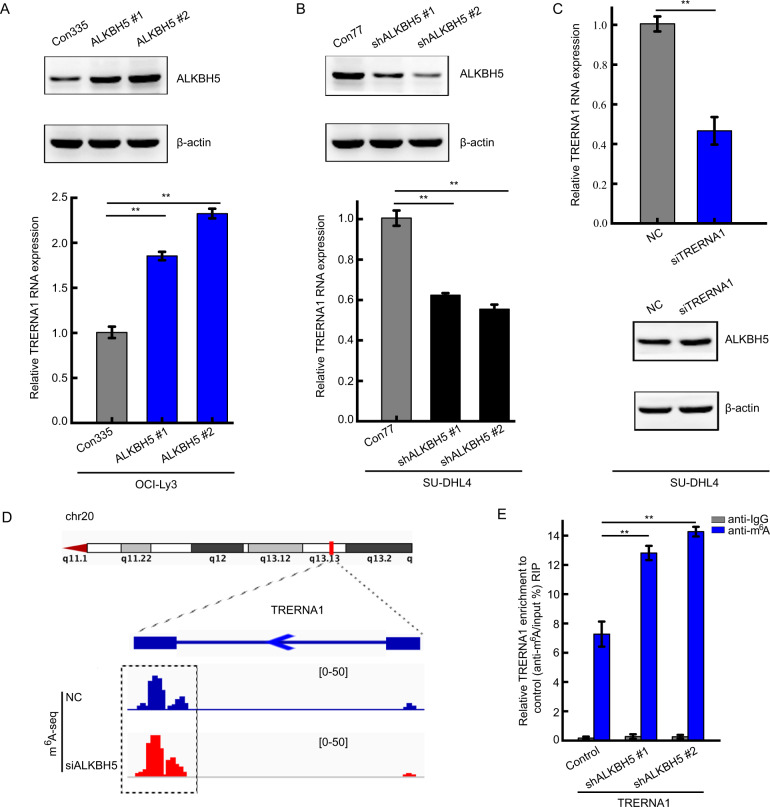
ALKBH5 upregulates TRERNA1 expression via m^6^A modification. **A** TRERNA1 levels in OCI-Ly3 stable ALKBH5 overexpression and control cells were verified. **B** TRERNA1 levels in SU-DHL4 stable ALKBH5 knockdown and control cells were verified. **C** ALKBH5 levels were verified by western blot analysis after transfecting cells with NC or siTRERNA1 for 48 h. **D** m^6^A levels of TRERNA1 were measured in ALKBH5-silenced cells by MeRIP-seq. **E** MeRIP-qPCR results verified the TRERNA1 m^6^A modification upon ALKBH5 silencing. The data are presented as the mean ± SD. **P < 0.01.

m^6^A modification has been shown to multiply modulate lncRNA processes, such as nuclear export, translation, and RNA decay [21, 22]. Given that ALKBH5 was previously reported to regulate RNA degradation as a key m^6^A demethyltransferase, we next assessed whether ALKBH5 can regulate TRERNA1 expression by m^6^A modification. We observed that TRERNA1 was upregulated by ALKBH5 overexpression in OCI-Ly3 cells (Fig. 3A). In contrast, ALKBH5 knockdown resulted in a remarkable decrease in TRERNA1 expression in SU-DHL4 cells (Fig. 3B). Nevertheless, TRERNA1 knockdown had no effect in the expression of ALKBH5 (Fig. 3C and Fig. S1B). To further elucidate whether TRERNA1 is regulated by ALKBH5-mediated m^6^A methylation, we performed methylated RNA immunoprecipitation sequencing (MeRIP-seq) combined with RNA sequencing to compare the global m^6^A target gene profiles between ALKBH5-depleted and control SU-DHL4 cells. The MeRIP-seq data showed that the m^6^A levels of TRERNA1 were increased when ALKBH5 was silenced (Fig. 3D). Moreover, we designed MeRIP experiment to detect the m^6^A modification of TRERNA1 by ALKBH5. ALKBH5 deficiency substantially increased the m^6^A level on TRERNA1, indicating that ALKBH5 is a major m^6^A demethylase for TRERNA1 in SU-DHL4 cells (Fig. 3E). The results showed that ALKBH5 upregulated lncRNA TRERNA1 expression via an m^6^A methylation-dependent mechanism.

### m^6^A modification decreases TRERNA1 transcript stability

**Fig. 4:**
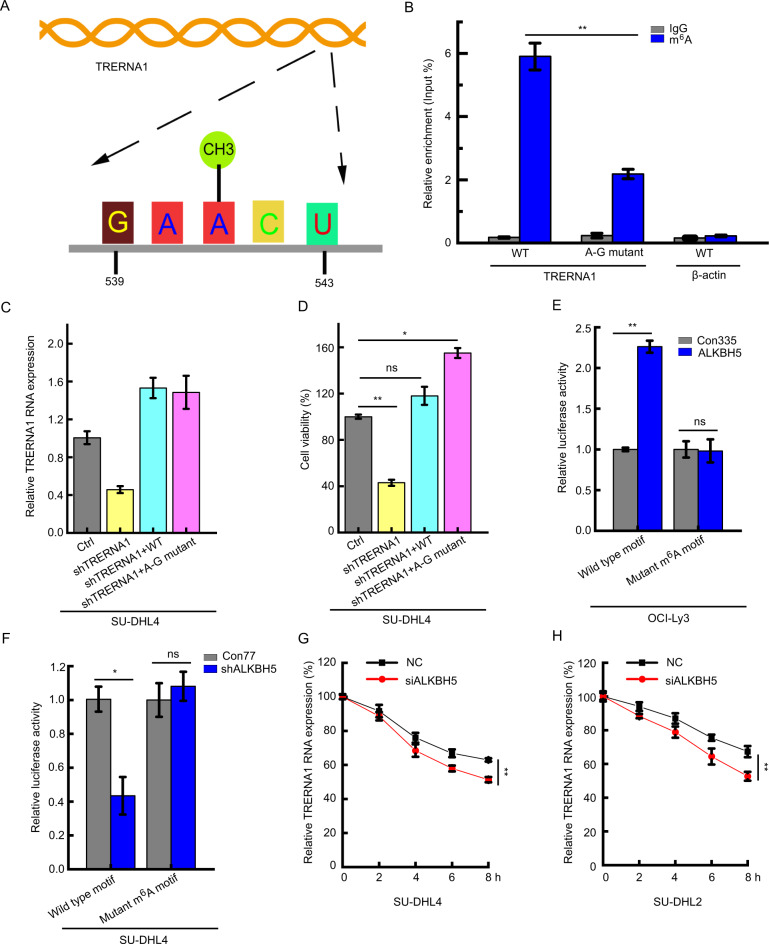
m^6^A enrichment is negatively associated with TRERNA1 activity in DLBCL. **A** Schematic representation of the position of m^6^A motifs within TRERNA1. **B** Changes in m^6^A-modified TRERNA1 levels between wild-type and mutants in SU-DHL4 cells; A-G mutant, mutant with A-G transition mutations. **C** TRERNA1 expression in control and shTRERNA1 SU-DHL4 cells in the presence of the indicated overexpression constructs. **D** Cell viability in the shControl- and shTRERNA1-treated SU-DHL4 cells in the presence of the indicated overexpression constructs. **E** Relative luciferase activity of TRERNA1 with either wild-type or A–G mutant m^6^A sites after the transfection of OCI-Ly3 cells with LV-ALKBH5. **F** Relative luciferase activity of TRERNA1 with either wild-type or A–G mutant m^6^A sites after the transfection of SU-DHL4 cells with LV-shALKBH5. **G**, **H** TRERNA1 levels in siALKBH5 and control cells were quantified by qRT-PCR at the indicated time points after actinomycin D treatment. The cells were treated with 5 μg/ml of actinomycin D at 0, 2, 4, 6, and 8 h. The data are presented as the mean ± SD; *n* = 3. *P < 0.05, **P < 0.01. ns nonsignificant.

To further evaluate the potential effects of m^6^A modification of TRERNA1, we analyzed the m^6^A residues located in TRERNA1 5′-DRACH-3′ motif (D = A/G/U, R = G/A, and H = C/A/U) (Fig. 4A). Next, we mutated m^6^A motifs in TRERNA1 in which the adenine residues were replaced with guanine (A-G transition mutation, A541-G). As expected, m^6^A modification of TRERNA1 was drastically decreased when m^6^A motifs were mutated, as assessed by MeRIP assays. These results confirmed that the mutated motif is the site of m^6^A modification in TRERNA1 (Fig. 4B). To assess the function of m^6^A modification of TRERNA1, we transfected the wild-type or m^6^A A-G transition mutated TRERNA1 or the control into TRERNA1 knockdown cells. TRERNA1 expression was restored in shTRERNA1-treated cells following the introduction of either the wild-type or mutant TRERNA1, as assessed by RT-qPCR (Fig. 4C). However, compared to the wild-type TRERNA1, overexpression of the A-G mutant resulted in a higher cell proliferation rate (Fig. 4D). These results showed that a low level of TRERNA1 m^6^A methylation is necessary to promote DLBCL cell proliferation. We next performed luciferase reporter assays to assess the requirement for the m^6^A modification of TRERNA1 for its regulation by ALKBH5. Luciferase activity was higher in ALKBH5 upregulation OCI-Ly3 cells transfected with wild-type TRERNA1 than the control, while mutations in the m^6^A sites abrogated this upregulation (Fig. 4E). We also found that ALKBH5 knockdown decreased the activity of the luciferase in wild-type construct containing TRERNA1, while TRERNA1 at A-G mutation rendered resistance to the effect of ALKBH5 knockdown (Fig. 4F). We then investigated whether m^6^A modification can affect the RNA stability of TRERNA1. For RNA half-life profiling, ALKBH5-silenced cells were treated with the transcriptional inhibitor actinomycin D at 5 µg/ml for various times. As shown in Fig. 4G, H, the loss of ALKBH5 significantly shortened the half-life of TRERNA1. Taken together, these results suggested that ALKBH5 functions as an eraser for methylated TRERNA1 to increase its stability.

### m^6^A modification of TRERNA1 epigenetically silences p21 transcription by promoting its interaction with EZH2

**Fig. 5:**
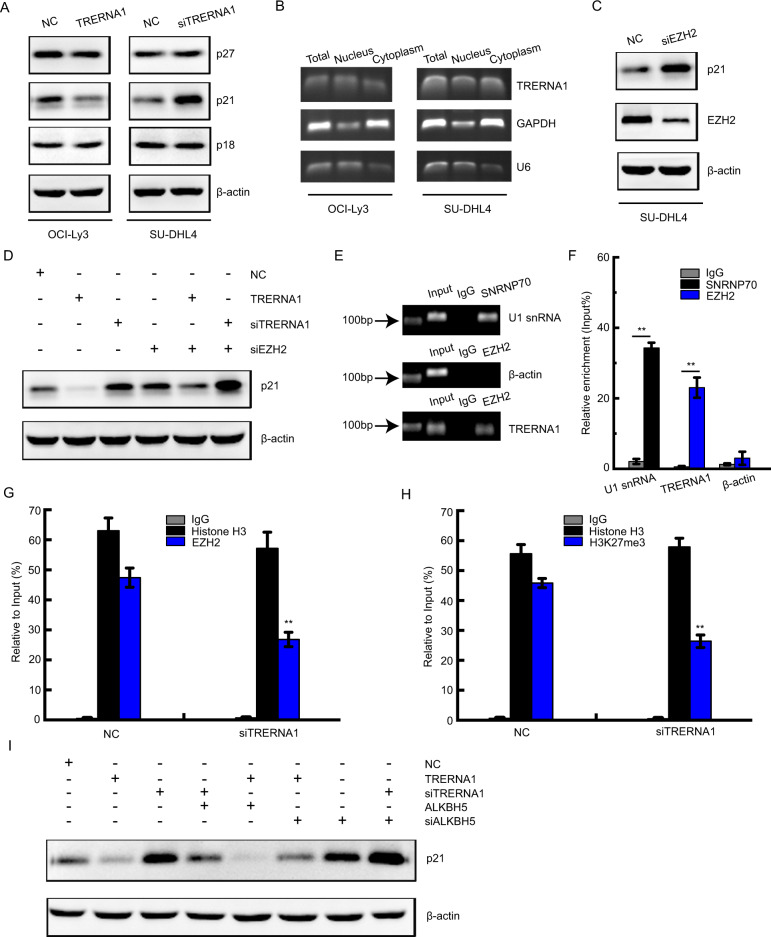
m^6^A modification of TRERNA1 epigenetically silences p21 transcription by interacting with EZH2. **A** The protein levels of cyclin-dependent kinases inhibitors (CDKIs) were detected after TRERNA1 overexpression or downregulation. **B** The subcellular fractions of TRERNA1 were analyzed by RT-PCR. U6 and GAPDH were used as controls for the nuclear and cytoplasmic fractions, respectively. **C** The protein levels of p21 were detected after EZH2 downregulation in SU-DHL4 cells. **D** The protein levels of p21 were detected when knockdown of TRERNA1 and EZH2 collectively. **E** RIP assays were performed to verify the enrichment of EZH2 on TRERNA1 in SU-DHL4 cells by gel electrophoresis. SNRNP70 and IgG were used as positive and negative controls, respectively. **F** TRERNA1, U1 snRNA and β-actin from the RIP assay were also analyzed by RT-qPCR. **G** ChIP assays were performed to evaluate the effects of EZH2 on binding the p21 gene promoter upon TRERNA1 downregulation in SU-DHL4 cells. **H** ChIP assays were performed to evaluate the effects of H3K27me3 modification of the p21 gene promoter upon TRERNA1 downregulation in SU-DHL4 cells. Histone H3 and IgG were used as positive and negative controls, respectively. **I** The expression of p21 was assessed by western blot analysis in cells transfected with ALKBH5 or siALKBH5. The data are presented as the mean ± SD; *n* = 3. **P < 0.01.

Cyclin-dependent kinases inhibitors (CDKIs) play key roles in the G1/S phase transition. To investigate the mechanism of cell cycle dysregulation, we next assessed the protein levels of CDKIs such as p18, p21, and p27 upon TRERNA1 treatment. The results showed that TRERNA1 overexpression downregulated p21 expression without affecting p18 and p27 levels in OCI-Ly3 cells. Consistent with these results, TRERNA1 knockdown in SU-DHL4 cells promoted p21 expression (Fig. 5A). Among the assayed genes, p21 was selected for further investigation. To elucidate the molecular mechanism associated with TRERNA1-mediated regulation, we next evaluated the subcellular localization of TRERNA1. We observed that TRERNA1 was localized in both the cytoplasm and the nucleus of DLBCL cells, suggesting that it functions in both the cytoplasm and nucleus (Fig. 5B). Previous studies have shown that lncRNAs, including UCA1, TUG1, and XIST can recruit EZH2 to epigenetically silence the expression of CDKIs [23]. EZH2, a core subunit of methyltransferase polycomb repressive complex 2 (PRC2), can catalyze the trimethylation of lysine residue 27 of histone 3 (H3K27me3) to epigenetically modulate target gene expression. We observed that EZH2 knockdown significantly increased p21 expression in DLBCL cells, similar to TRERNA1 (Fig. 5C). Knockdown of TRERNA1 and EZH2 collectively enhanced the expression of p21 (Fig. 5D). Therefore, we hypothesized that TRERNA1 may inhibit p21 expression by interacting with EZH2. RNA immunoprecipitation assay results confirmed that TRERNA1 could directly bind to EZH2 (Fig. 5E, F), demonstrating a specific association between EZH2 and TRERNA1. Next, we conducted chromatin immunoprecipitation (ChIP) assays to evaluate the effects of TRERNA1 on EZH2 binding and H3K27me3 modification of the p21 gene promoter in SU-DHL4 cells. As expected, TRERNA1 knockdown decreased the EZH2 enrichment and H3K27me3 modification of the p21 promoter compared to that observed in the control (Fig. 5G, H and Fig. S3A, B). SUZ12 is a key component of PRC2, we also found that TRERNA1 could interact with SUZ12 to repress p21 expression (Fig. S4). To assess whether ALKBH5 was responsible for the observed EZH2-mediated expression of p21, we transfected siALKBH5 or ALKBH5 overexpression constructs into OCI-Ly3 cells. Overexpressed ALKBH5 further inhibited p21 protein expression (Fig. 5I). We further determined whether p21 functioned as a key downstream in TRERNA1-involved DLBCL cell proliferation. As shown in Fig. S5A, we found that p21 downregulation significantly reversed the inhibition of cell proliferation induced by silencing TRERNA1. Moreover, upregulated p21 obviously abolished the proliferation induced by TRERNA1 overexpression. There is a negative relationship between TRERNA1 and p21 expression levels in our lymphoma tissues (Fig. S5B). These results indicated that low levels of m^6^A modification in TRERNA1 can epigenetically silence p21 expression by interacting with EZH2 to promote cell proliferation.

### TRERNA1 promotes tumorigenesis of DLBCL in vivo

**Fig. 6:**
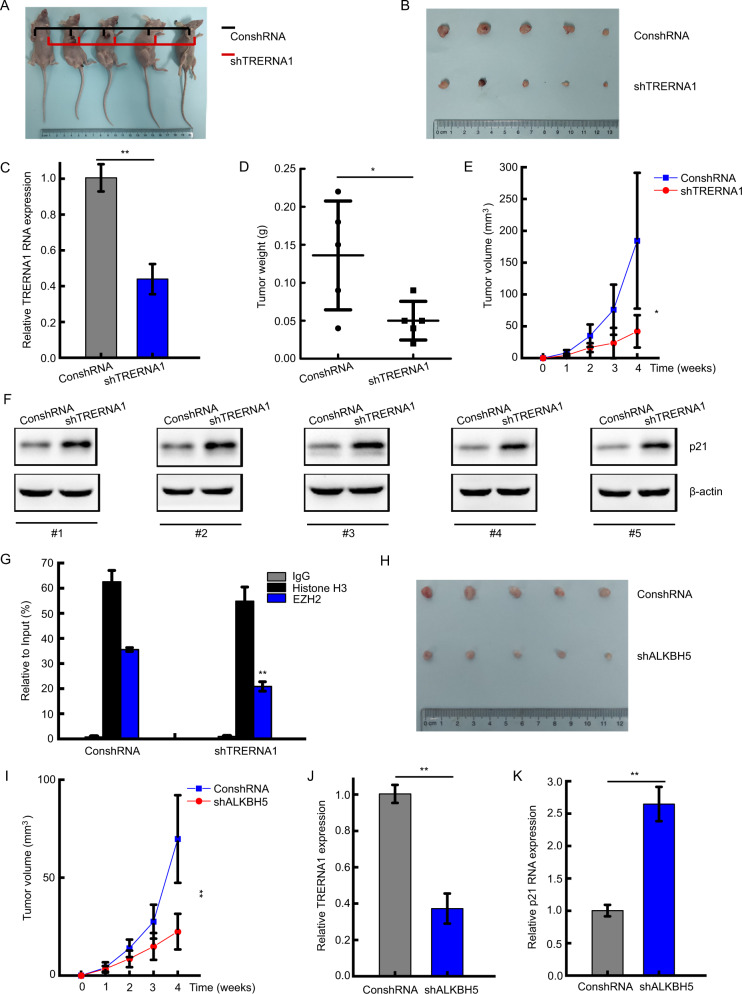
TRERNA1 promotes DLBCL tumorigenesis in vivo. **A**, **B** Tumor growth model showed that BALB/c nude mice were injected with stable shTRERNA1 knockdown and control SU-DHL4 cells via subcutaneous injection. **C** TRERNA1 expression in tumors detected by RT-qPCR. **D** Weights of the formed tumors. **E** Tumor volume growth curves. **F** p21 expression in tumors detected by western blot analysis. **G** The enrichment of EZH2 at the p21 promoter when TRERNA1 was decreased in the xenograft. **H** Tumor growth model showed that BALB/c nude mice were injected with stable ALKBH5 knockdown and control SU-DHL4 cells via subcutaneous injection. **I** Tumor volume growth curves with stable ALKBH5 knockdown and control SU-DHL4 cells. **J** TRERNA1 expression in tumors detected by qRT-PCR. **K** p21 expression in tumors detected by qRT-PCR. The data are presented as the mean ± SD; *P < 0.05, **P < 0.01.

We subsequently confirmed the ability of TRERNA1 to promote the tumorigenesis of DLBCL in vivo. To this end, we subcutaneously injected BALB/c nude mice with stable shTRERNA1 knockdown and control SU-DHL4 cells. At 4 weeks after the injection, the tumor growth models showed that TRERNA1 knockdown significantly inhibited tumor growth (Fig. 6A, B). We also observed that the level of TRERNA1 expression was decreased in the shTRERNA1 group derived from tumor tissues by RT-qPCR assay (Fig. 6C). At the end of the experiment, the mean weights of tumors from mice in the shTRERNA1 group were markedly lower than those in the control vector group (Fig. 6D). As shown in Fig. 6E, the volumes of xenograft tumors in the shTRERNA1 group were much lower than those of the control vector group. Western blot results confirmed that TRERNA1 knockdown upregulated p21 expression in the xenograft models (Fig. 6F). Moreover, we detected that the enrichment of EZH2 at the p21 promoter was decreased in the xenograft when TRERNA1 was downregulated, which confirmed the TRERNA1-mediated p21 inhibition (Fig. 6G). As shown in Fig. 6H, I, the tumor growth models showed that ALKBH5 knockdown significantly inhibited tumor growth. TRERNA1 expression was decreased in the shALKBH5 group derived in vivo (Fig. 6J). On the contrary, p21 expression was upregulated in the shALKBH5 group (Fig. 6K). These data indicated that lncRNA TRERNA1 functions as an oncogene by downregulating p21 expression in DLBCL.

## Discussion

DLBCL is the most common form of B cell non-Hodgkin lymphoma (B-NHL), accounting for ~40% of all B-NHL diagnoses comprising a range of phenotypically, genetically, and clinically distinct malignancies [24]. Epigenetic modification has been shown to drive tumorigenesis and pathogenesis in most hematological malignancies, and some drugs have been designed to counteract aberrant DNA methylation, histone acetylation, and histone methylation for epigenetic therapy [4, 25, 26]. However, identifying novel biomarkers to guide DLBCL therapy remained a major challenge [19].

The molecular heterogeneity of DLBCL has been demonstrated at the mRNA and miRNA levels [27]. In recent years, lncRNA dysregulation has emerged as a crucial process in the initiation and progression of cancer [9, 28], with lncRNA TRERNA1 having been shown to regulate transcriptional activity in a *cis* or *trans* manner to promote cell invasion and metastasis in breast cancer, HCC, and ependymoma [12, 14, 29]. However, the roles of lncRNAs in DLBCL have yet to be elucidated. In the present study, we showed that lncRNA TRERNA1 exhibits higher expression in DLBCL tissues than in normal lymph node hyperplasia tissues and can promote cell proliferation in vitro and in vivo. Our results demonstrate that TRERNA1 functions as an oncogene in DLBCL progression.

m^6^A dysregulation has been shown to play an important role in the carcinogenesis and metastasis of human malignancies [30]. However, the functions and molecular mechanisms of m^6^A in the modification of ncRNAs remain largely unknown. While investigating the potential role of the m^6^A modification of TRERNA1 in lymphoma, we first observed that m^6^A levels were decreased in DLBCL tissues. Epigenetic m^6^A modification is modulated by regulators such as methyltransferases, demethylases, and reading enzymes [31]. Our results showed that the demethylase ALKBH5 promotes TRERNA1 expression. Our study also showed that the clinical significance of ALKBH5 and TRERNA1 in DLBCL. ALKBH5 functions as a well-known demethylase to reverse m^6^A methylation and plays a crucial oncogenic role in sustaining tumorigenicity [32, 33]. For instance, ALKBH5 has been shown to maintain the tumorigenicity of glioblastoma stem-like cells by sustaining FOXM1 expression and cell proliferation program [34]. ALKBH5 inhibits the autophagy of epithelial ovarian cancer cells through miR-7 and BCL-2 [35]. However, whether ALKBH5 can regulate lncRNA expression through m^6^A modifications in DLBCL has remained unelucidated.

It has remained unclear whether TRERNA1 is modified by m^6^A methylation [36]. In the present study, we showed that TRERNA1 can be modified by ALKBH5 and observed that ALKBH5 upregulation promotes TRERNA1 expression. These results were supported by MeRIP-seq results showing that the m^6^A levels of TRERNA1 increased when ALKBH5 was silenced. Moreover, MeRIP-qPCR results showed that the m^6^A methylation levels of TRERNA1 were upregulated upon ALKBH5 silencing. Strikingly, m^6^A mutations of TRERNA1 were observed to increase cell proliferation. m^6^A modification can have different effects on RNA stability [37, 38]. In our present study, we also observed that the loss of ALKBH5 significantly decreased TRERNA1 expression. Our results showed that the ALKBH5-mediated reduction in the m^6^A methylation of TRERNA1 is necessary to promote DLBCL cell proliferation.

The cell cycle is regulated by CDKIs such as p18, p21, and p27 [39, 40]. We observed that p21 protein levels were decreased upon TRERNA1 treatment. As a core component of PRC2, EZH2 has been reported to epigenetically silence gene expression by catalyzing H3K27me3 modification in various malignancies [41, 42]. Thus we hypothesized that TRERNA1 epigenetically regulated p21 through EZH2. Interestingly, we observed that TRERNA1 knockdown decreased EZH2 binding and H3K27me3 modification levels across the p21 promoter via RIP and ChIP assays. Furthermore, m^6^A demethylase ALKBH5 further reduced p21 expression. TRERNA1 was also shown to inhibit p21 expression to promote tumorigenesis in vivo.

**Fig. 7:**
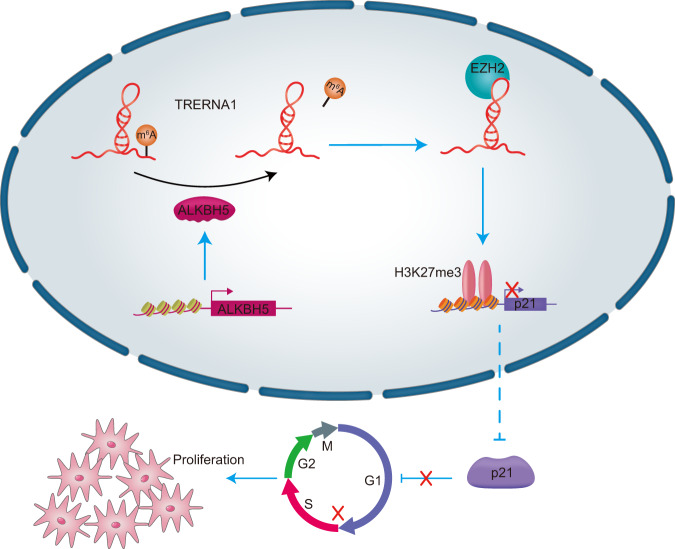
A schematic diagram of lncRNA TRERNA1 functions in DLBCL. ALKBH5-mediated upregulation of TRERNA1 with a low level of m^6^A modification can promote cell proliferation and cell cycle progression. The m^6^A modification of TRERNA1 was shown to epigenetically silence p21 transcription to accelerate cell cycle progression by interacting with EZH2.

In summary, the results of the present study show that TRERNA1 is a novel lncRNA biomarker in DLBCL. A positive relationship between ALKBH5 and TRERNA1 expression was demonstrated, and we observed that the ALKBH5-mediated upregulation of TRERNA1 with a low level of m^6^A modification can promote cell proliferation and cell cycle progression. Interestingly, the m^6^A modification of TRERNA1 was shown to epigenetically silence p21 transcription to accelerate cell cycle progression by interacting with EZH2 (Fig. 7). Based on our results, the m^6^A modification of TRERNA1 may not only have an important role in DLBCL progression but also serve as a prognostic indicator for therapeutic intervention.

## Materials and methods

### Cell lines and culture

The human cell lines SU-DHL4 and OCI-Ly3 were purchased from the American Type Culture Collection (ATCC, Manassas, VA, USA) and cultured in RPMI-1640 medium supplemented with 10% fetal bovine serum, 100 U/ml penicillin, and 100 μg/ml streptomycin (Invitrogen, Carlsbad, CA, USA) under an atmosphere with 5% CO_2_ at 37 °C.

### Tissue samples

Tissue specimens from resected lymphoma, DLBCL lymph gland, and normal lymph gland biopsies were collected between 2018 and 2020 from Nanjing Drum Tower Hospital. All tissues were immediately stored at −80 °C. Informed consent was obtained from each patient, and ethical consent was granted by the Medical Ethics Committee of the Affiliated Hospital of Nanjing University Medical School.

### Plasmid construction and cell transfection

The lncRNA TRERNA1 cDNA plasmid was constructed by GENEWIZ (Suzhou, China). In addition, vector-based short hairpin RNAs (shRNAs) against TRERNA1 and scrambled sequences (used as control shRNAs) were constructed. The ALKBH5, shALKBH5, or empty vector lentiviral plasmids were purchased from GeneChem (Shanghai, China). Cell lines exhibiting stable ALKBH5 overexpression or silencing were selected by puromycin (2 μg/ml) for 2 weeks at 48 h after transfection. Small interfering RNAs (siRNAs) were synthesized by GenePharma (Shanghai, China). Cells were transfected with siRNAs utilizing Lipofectamine 2000 (Invitrogen, Carlsbad, CA, USA). After transfection for 48 h, RT-qPCR and western blot analyses were performed to verify the transfection efficiency. The sequences of the siRNAs against specific targets assayed in this study are listed in Supplemental Table S1.

### Quantitative RT-PCR

Total RNA was extracted from cells or frozen tissues using TRIzol reagent (Invitrogen, Carlsbad, CA, USA) and used as a template to synthesize cDNA with a reverse transcription kit (Takara, Dalian, China). Quantitative reverse transcription PCR (RT-qPCR) was performed using SYBR Green reagents (Takara, Dalian, China), with β-actin used as an internal control. The primers used in the present study are listed in Supplemental Table S2.

### Western blot analysis

The protein concentration of cell lysates was quantified using a bicinchoninic acid protein assay kit (Beyotime, Jiangsu, China). Equal amounts of protein were analyzed by immunoblotting using an anti-ALKBH5 antibody (#ABE547, Millipore, CA, USA), an N^6^-methyladenosine (m^6^A) (D9D9W) Rabbit mAb (#56593S, CST, MA, USA), and a p21 Waf1/Cip1 (12D1) Rabbit mAb (#2947, CST, MA, USA), with an anti-β-actin antibody (Sigma-Aldrich, USA) used as an internal control.

### Cell proliferation assay

A Cell Counting Kit-8 (CCK-8, Dojindo, Kumamoto, Japan) was used to assess cell proliferation. SU-DHL4 and OCI-Ly3 cells were seeded into 96-well plates (3×10^3^ cells per well), and CCK-8 reagent was added to the wells at 0, 24, 48, 72. or 96 h. Subsequently, the absorbance values of the samples were measured at 450 nm.

### Cell cycle analysis

Cells were first harvested after 72 h of transfection and the cells were washed twice with PBS containing 1% fetal bovine serum and incubated in PBS containing 0.02% TritonX-100, 0.1 mg/ml RNase (Sigma-Aldrich), and 10 μg/ml propidium iodide (PI, 40%, Sigma-Aldrich) for 30 min at 37 °C. The cell cycle was detected by a FACScan flow cytometer (Becton Dickinson & Co., San Jose, CA, USA).

### Xenograft tumor models

Xenograft tumor models were generated in BALB/c nude mice (4 weeks old), which were purchased from the Model Animal Research Center of Nanjing Medical University (Nanjing, China). The experimental protocol was approved by the Nanjing Drum Tower Hospital. The experiments conformed to sample size estimate, randomization, and blinding for animal studies. Each nude mouse was subcutaneously injected with 2 × 10^6^ cells resuspended in 200 μl PBS (*n* = 5 per group). Then, the tumor growth rate and volume were measured each week, with the latter measured by calculating the length (*L*) and width (*W*) diameter of the tumor using the following formula: *V* = 0.5 × *L* × *W*^2^. After 4 weeks, the mice were sacrificed, and xenografts were removed for volume and weight measurements.

### RNA m^6^A methylation quantification

The total m^6^A levels of extracted RNA were measured using an EpiQuik^TM^ m^6^A RNA Methylation Quantification Kit (Colorimetric, Epigentek, NY, USA) following the manufacturer’s instructions. For each sample, 200 ng of poly-A-purified RNA was coated onto assay wells, and the m^6^A levels were quantified by measuring the absorbance of each sample at 450 nm.

### RNA immunoprecipitation (RIP)

A Magna MeRIP^™^ m^6^A Kit (Millipore, Catalog No. 17-10499) was used for RNA m^6^A immunoprecipitation (RIP) according to the manufacturer’s instructions. An m^6^A antibody (#56593S, CST, MA, USA) was used to pull down m^6^A-modified lncRNA TRERNA1. Total RNA was isolated from cells using TRIzol reagent following the manufacturer’s instructions. Then, 10 μg of the anti-m^6^A antibody or anti-IgG was bound to protein A/G magnetic beads to immunoprecipitate m^6^A-modified RNA. The antibody against EZH2 used for RIP assays was purchased from Abcam (ab191250). Subsequently, RNA that was eluted and purified from the beads was quantified by qRT-PCR [43].

### MeRIP sequencing

Total RNA was extracted from cells using TRIzol reagent (Invitrogen, Carlsbad, CA, USA). Briefly, 10% of the fragmented RNA was used as the input sample, and the remaining RNA was incubated with the anti-m^6^A antibody using a Magna MeRIP^™^ m^6^A kit (Millipore, Catalog No. 17-10499) following the manufacturer’s instructions [44]. Then, MeRIP RNA was analyzed by deep sequencing on an Illumina Novaseq^™^ 6000 platform at the Lian-Chuan Genomic Facility (LC-bio, Hangzhou, Zhejiang, China) [45, 46].

### Dual-luciferase reporter assay

Luciferase reporter constructs were generated by cloning wild-type or mutant TRERNA1 (RiboBio, Guangzhou, China), and A-G mutation of the TRERNA1 reporter plasmid was performed using a QuikChange Lightning Multi Site-Directed Mutagenesis kit (Agilent Technologies). Cell lysates were harvested 48 h after transfection, and luciferase activities were detected using the Dual-Luciferase Reporter Assay System (Promega, Madison, WI, USA).

### mRNA stability

TRERNA1 RNA stability was measured after ALKBH5 knockdown in SU-DHL4 and OCI-Ly3 cells treated with 5 μg/ml of actinomycin D (Act-D, #A9415, Sigma, MO, USA) for the indicated time periods. TRERNA1 was measured by RT-qPCR after incubation. The RNA half-life (t1/2) of TRERNA1 was calculated using the equation ln2/slope with β-actin used for normalization [47].

### Chromatin immunoprecipitation (ChIP) assays

ChIP assays were performed using an EZ ChIP^™^ Chromatin Immunoprecipitation kit (Millipore, USA) following the manufacturer’s instructions. Briefly, the crosslinked chromatin DNA was sonicated into 200-700 bp fragments and then fixed with 1% formaldehyde. Immunoprecipitation was performed using anti-EZH2 (ab191250, Abcam), anti-H3K27me3 (#9733S, CST, MA, USA), anti-Histone H3 (positive control), and normal mouse IgG (negative control) antibodies. DNA was extracted and was used for qRT-PCR analyses. The specific primers used to amplify p21 are listed in Supplemental Table S2.

### Statistical analysis

An independent Student’s *t* test (two-tailed) was performed by comparing the means of a continuous variable between two groups. Origin 8.0 software was used to determine the correlation between two variables by Pearson’s correlation coefficient. The data are presented as the mean ± SD. Differences were considered significant at *P*-value < 0.05 (*P < 0.05, **P < 0.01). The error bars represent the mean ± SD (ns indicates that the difference is not significant).

## Supplementary information

Supplementary figure legends

Supplementary Tables

Supplementary Figure1

Supplementary Figure2

Supplementary Figure3

Supplementary Figure4

Supplementary Figure5

## Data Availability

All data generated or analyzed during this study are included in this published article and its Additional files.
