# Supplementary material for: ALKBH5-mediated N6-methyladenosine modification of TRERNA1 promotes DLBCL proliferation via p21 downregulation
Source: Cell Death Discov. 2022 Jan 14;8:25. doi: 10.1038/s41420-022-00819-7 (PMC8760254; doi:10.1038/s41420-022-00819-7)
Supplement: Supplementary file 1 — Supplementary figure legends [file 41420_2022_819_MOESM1_ESM.docx]

**Supplementary figure legends**

**Supplementary Table S1** **siRNA sequence of genes used in this manuscript**

**Supplementary Table S2 Primers used for reverse transcription and real-time PCR**

**Supplementary Fig. S1 The expression levels of TRERNA1 and ALKBH5.**

**A** qRT-PCR analysis of TRERNA1 expression in SU-DHL4 cells transfected with siRBM15 and siMETTL16. **B** ALKBH5 levels were verified by qRT-PCR analysis after transfecting cells with NC or siTRERNA1. The data are presented as the mean ± SD.

**Supplementary Fig. S2 The expression of TRERNA1 in DLBCL cells.**

**A** qRT-PCR analysis of relative expression of TRERNA1 in DLBCL cells.

**Supplementary Fig. S3 TRERNA1 represses transcription of p21 by recruiting EZH2 to the p21 promoter.**

**A** ChIP-PCR of EZH2 enrichment of the promoter region of the p21 after knockdown TRERNA1 in SU-DHL4 cells. **B** ChIP-PCR of H3K27me3 enrichment of the promoter region of the p21 after knockdown TRERNA1 in SU-DHL4 cells.

**Supplementary Fig. S4** **The interaction between TRERNA1 and SUZ12.**

**A** RIP assays were performed to verify the enrichment of SUZ12 on TRERNA1 in SU-DHL4 cells. SNRNP70 and IgG were used as positive and negative controls, respectively. **B** ChIP assays were performed to evaluate the effects of SUZ12 on binding the p21 gene promoter upon TRERNA1 downregulation in SU-DHL4 cells. The data are presented as the mean ± SD; *n* = 3. ***P* < 0.01.

**Supplementary Fig. S5** **The correlation between TRERNA1 and p21.**

**A** Cell proliferation of SU-DHL4 cells transfected with TRERNA1, siTRERNA1, p21 and si p21. **B** Scatter plot between TRERNA1 and p21 in DLBCL. The data are presented as the mean ± SD values; *n* = 3. **P* < 0.05, ***P* < 0.01.
