# Supplementary material for: ALKBH5-mediated N6-methyladenosine modification of TRERNA1 promotes DLBCL proliferation via p21 downregulation
Source: Cell Death Discov. 2022 Jan 14;8:25. doi: 10.1038/s41420-022-00819-7 (PMC8760254; doi:10.1038/s41420-022-00819-7)
Supplement: Supplementary file 2 — Supplementary Tables [file 41420_2022_819_MOESM2_ESM.docx]

**Supplementary Tables**

**Table S1** **siRNA sequence of genes used in this manuscript**

| Gene names Sequences | | |
| --- | --- | --- |
| Control-siRNA | Sense | GCUUCCUGCUACACAGAAUTT |
|  | Antisense | AUUCUGUGUAGCAGGAAGCTT |
| TRERNA1-siRNA | Sense | CCGAUUUGAGAGAGUGAGATT |
|  | Antisense | UCUCACUCUCUCAAAUCGGTT |
| ALKBH5-siRNA | Sense | UCAACAGCGCCGUCAUCAATT |
|  | Antisense | UUGAUGACGGCGCUGUUGATT |
| EZH2-siRNA | Sense | GAGGUUCAGACGAGCUGAUUU |
|  | Antisense | AAAUCAGCUCGUCUGAACCUC |
| METTL3-siRNA | Sense | GCAAGUAUGUUCACUAUGATT |
|  | Antisense | UCAUAGUGAACAUACUUGCTT |
| METTL14-siRNA | Sense | GGACUUGGGAUGAUAUUAUTT |
|  | Antisense | AUAAUAUCAUCCCAAGUCCTT |
| WTAP-siRNA  FTO-siRNA | Sense  Antisense  Sense  Antisense | GCUUUGGAGGGCAAGUACATT  UGUACUUGCCCUCCAAAGCTT  CUAGGGUUUUGCUUCCAGAATT  UUCUGGAAGCAAAACCCUAGTT |

**Table S2** **Primers used for reverse transcription and real-time PCR**

| Primer names Sequences | | | | |
| --- | --- | --- | --- | --- |
| TRERNA1 | Forward(5’-3’) | | CCGTTGGCTCCACAAACCT | |
|  | Reverse (5’-3’) | | CAGTGACAGTAGCAGGCATCCT | |
| β-actin | Forward(5’-3’) | | GTCATTCCAAATATGAGATGCGT | |
|  | Reverse (5’-3’) | | GCTATCACCTCCCCTGTGTG | |
| ALKBH5 | Forward(5’-3’) | | CCCGAGGGCTTCGTCAACA | |
|  | Reverse (5’-3’) | | CGACACCCGAATAGGCTTGA | |
| EZH2 | Forward(5’-3’) | | TGCACATCCTGACTTCTGTG | |
|  | Reverse (5’-3’) | | AAGGGCATTCACCAACTCC | |
| U6 | Forward(5’-3’) | | CGCTTCGGCAGCACATATACTA | |
|  | Reverse (5’-3’) | | CGCTTCACGAATTTGCGTGTCA | |
| GAPDH | Forward(5’-3’) | | GCACCGTCAAGGCTGAGAAC | |
|  | Reverse (5’-3’) | | TGGTGAAGACGCCAGTGGA | |
| p21 | Forward(5’-3’)  Reverse (5’-3’) | | GAGACTCTCAGGGTCGAAAACG  GGATTAGGGCTTCCTCTTGGA | |
| ChIP p21 | Forward(5’-3’)  Reverse (5’-3’) | | CTGCCTCTGCTCAATAATGTTCT  GGAATTCACCTTCACACAGGC | |
|  | |  | |  |
